# On Crowns

**Published:** 1893-06

**Authors:** David Genese

**Affiliations:** Baltimore, Md.


### ON CROWNS.[1]

BY DAVID GENESE, D.D.S., BALTIMORE, MD.

Crowning and fronting teeth, of which to-day's clinic was a practical demonstration, has been in vogue many years. Names of prominent dental practitioners have been from time to time associated with new ideas upon the subject, and I have only submitted for your consideration eclectic points culled from the different methods known to us to simplify our work, and used with greater ease to the patient, precision in adjusting, natural appearance, and

---

[1] Read before the Odontological Society of Pennsylvania, April 8, 1893.

lastly, though not the least, with less destruction to the root and its organism.

My earliest recollection of this class of work is a natural tooth-crown, drilled through and countersunk like the Bonwill, affixed to the root by a leaden pivot pressed into the root, previously shaped to hold the lead, which pressed through the natural tooth and was hammered tightly in its place.

Work of this character is illustrated by specimens presented by me to the Maryland University.

The introduction of porcelain teeth produced a marked improvement in this branch of dentistry, and brought to us many methods of crowning, such as the Howe, Gates, Bonwill, Rynear, and numerous gold-crown systems. The latter I leave to the good judgment of the admirers of a natural appearance in the mouth to decide their merits. A free edge near the gingival border is objectionable, while the destruction of the attachment of the gum margin to the root is a serious defect.

What I endeavored to obtain by the method shown to-day is conservation of the root with its marginal covering, also to avoid the necessity of reaming or drilling the pulp-canal to a given size to fit a pivot already formed irrespective of the conditions that might be found, and to adjust a bifurcated pivot so exactly that no undue strain should be the result to cause irritation after the crown was in place, and to have the pivot so under control that should a curve be in the root it will adapt itself to it, avoiding that distressing cause of so much trouble in pivoting,—pressing the sides of the root into the alveolar process, with its train of troubles.

To the gentlemen who were not at the clinic I would briefly state that these crowns are made to conform as nearly as possible to the natural roots found in a number of models, with sufficient porcelain left on them to allow for grinding in any direction, their density permitting them to be repolished.

A cup of irido-platinum is burnt into them, which has a head pressed upon it to prevent pulling out. It can be subjected to heat sufficient to melt pure gold without fear of cracking, as the tubular form allows it to shrink indefinitely, making at each firing a tighter hold on the platinum lining, as shown in specimens passed around.

The pivot is of the same platinum, formed from the sheet, and left hollow to allow for fitting into the canal its entire length. This can be kept hollow by plugging with asbestos or made solid by drawing the solder into it by the flux.

By this system you will see that the pivot and crown are independently fitted to the root, and when soldered will occupy exactly their relative positions to each other, requiring very little cement to hold them in place.

Apart from this, each step of the work is under close inspection with mirror and models and by the point of instruments to detect any overlap, and the extra length of porcelain allows for grinding, fitting, and placing the crown at any angle independently of the pivot.

The soldering process is rendered safe and simple by the use of the little holder of pin metal and Teague's investing compound, as shown in specimens, which holds the post and tooth in position. The flame should be directed well onto the body of the tooth in soldering, rendering it the hottest part when solder flows, and the specimen in sections will demonstrate the union of tooth and pivot.

The higher the standard of solder used the better the result, as platinum cannot be properly soldered with poor metal.

Pure silver or gold or 18-carat plate gives the best result.

For accuracy of adjusting, models are taken after the root is prepared; the crown is fitted and articulated, and tried in its place, the hollow is filled with Parr's wax flux, which prevents grit from the wheels or any substance being in the platinum cup that might interfere with soldering. The pivot in its place, and the crown already proved correct, warm the wax and adjust to position, then cool. When withdrawn, invest as shown by model and solder. Ten minutes after, the crown is ready to place in position.

The usual precaution of sealing the apex and drying the canal is needed. Gutta-percha is good in some cases, but I prefer fossiline, imported from Europe. It wears well, is a safe and easily-worked oxyphosphate, not setting too quickly, very adhesive and durable, while its chief advantage is intense hardness soon after fixing, and retaining its density, though mixed thinner than most of its similar compounds.

In finally adjusting the crown I place a wooden point on the automatic hammer and tap it a few times, forcing the crown to its closest adaptation and driving off the excess of cement.

It sets hard enough in ten minutes to allow grinding the crown, should the articulation require it.

If hurried from any cause, these crowns can be set by this cement without soldering, and will last several months. To those who prefer banding, this crown presents the strongest air-tight collar; no possibility of fluid entering between the metal and tooth, and the least amount of gold showing, as the edges are made flush with porcelain, as shown in specimen.

